# Crystal Structure of the Human Short Coiled Coil Protein and Insights into SCOC-FEZ1 Complex Formation

**DOI:** 10.1371/journal.pone.0076355

**Published:** 2013-10-01

**Authors:** Caroline Behrens, Beyenech Binotti, Carla Schmidt, Carol V. Robinson, John Jia En Chua, Karin Kühnel

**Affiliations:** 1 Department of Neurobiology, Max-Planck-Institute for Biophysical Chemistry, Göttingen, Germany; 2 Department of Chemistry, Physical and Theoretical Chemistry Laboratory, University of Oxford, Oxford, United Kingdom; Centro Nacional de Biotecnologia - CSIC, Spain

## Abstract

The short coiled coil protein (SCOC) forms a complex with fasciculation and elongation protein zeta 1 (FEZ1). This complex is involved in autophagy regulation. We determined the crystal structure of the coiled coil domain of human SCOC at 2.7 Å resolution. SCOC forms a parallel left handed coiled coil dimer. We observed two distinct dimers in the crystal structure, which shows that SCOC is conformationally flexible. This plasticity is due to the high incidence of polar and charged residues at the core *a/d*-heptad positions. We prepared two double mutants, where these core residues were mutated to either leucines or valines (E93V/K97L and N125L/N132V). These mutations led to a dramatic increase in stability and change of oligomerisation state. The oligomerisation state of the mutants was characterized by multi-angle laser light scattering and native mass spectrometry measurements. The E93V/K97 mutant forms a trimer and the N125L/N132V mutant is a tetramer. We further demonstrate that SCOC forms a stable homogeneous complex with the coiled coil domain of FEZ1. SCOC dimerization and the SCOC surface residue R117 are important for this interaction.

## Introduction

Human short coiled coil protein (SCOC) is an effector of the Golgi resident GTPase Arl1 [1] and was recently identified as a positive regulator of autophagy in a genome-wide siRNA screen [2]. The protein is widely expressed in the human body, most abundantly in the brain, heart and skeletal muscle [1].

SCOC interacts with fasciculation and elongation protein zeta 1 (FEZ1) [2-4]. Human FEZ1 (392 residues) is a mainly natively unfolded protein with three glutamate rich regions and a conserved coiled coil domain in the C-terminal half of the protein [5,6]. So far no structures are available for either SCOC or FEZ1. We are interested in the structural characterization of both proteins and the SCOC-FEZ1 complex in order to understand how they fulfill diverse biological functions.

FEZ1 acts as an adaptor in kinesin-1 mediated axonal transport to nerve terminals by binding to both the heavy chain of the motor protein kinesin-1 [7,8] and its cargo, for example as recently shown for Syntaxin 1a and Munc18 containing transport vesicles [9]. Phosphorylation of FEZ1 regulates cargo [10] and kinesin binding [9]. Mutations of the *C. elegans* FEZ1 orthologue UNC-76 lead to severe defects in axon growth and fasciculation as well as impaired axonal transport [9] [11]. A similar phenotype was observed when its binding partner UNC-69/SCOC was deleted, implying a role of the SCOC-FEZ1 complex for axonal outgrowth and normal presynaptic organization in *C. elegans* [4].

A distinct regulatory role in autophagy has also been attributed to SCOC and FEZ1 [2]. FEZ1 interacts with mammalian ULK1 kinase complex and its Drosophila orthologue [2,10]. The FEZ1-ULK1 complex inhibits autophagy induction, which is released upon binding of SCOC to FEZ1 [2]. SCOC-FEZ1 also forms a complex with UVRAG (UV radiation resistance associated gene). During starvation this complex dissociates and UVRAG binds to the Vps34 kinase complex instead, which in turn promotes autophagy [2].

**Figure 1:**
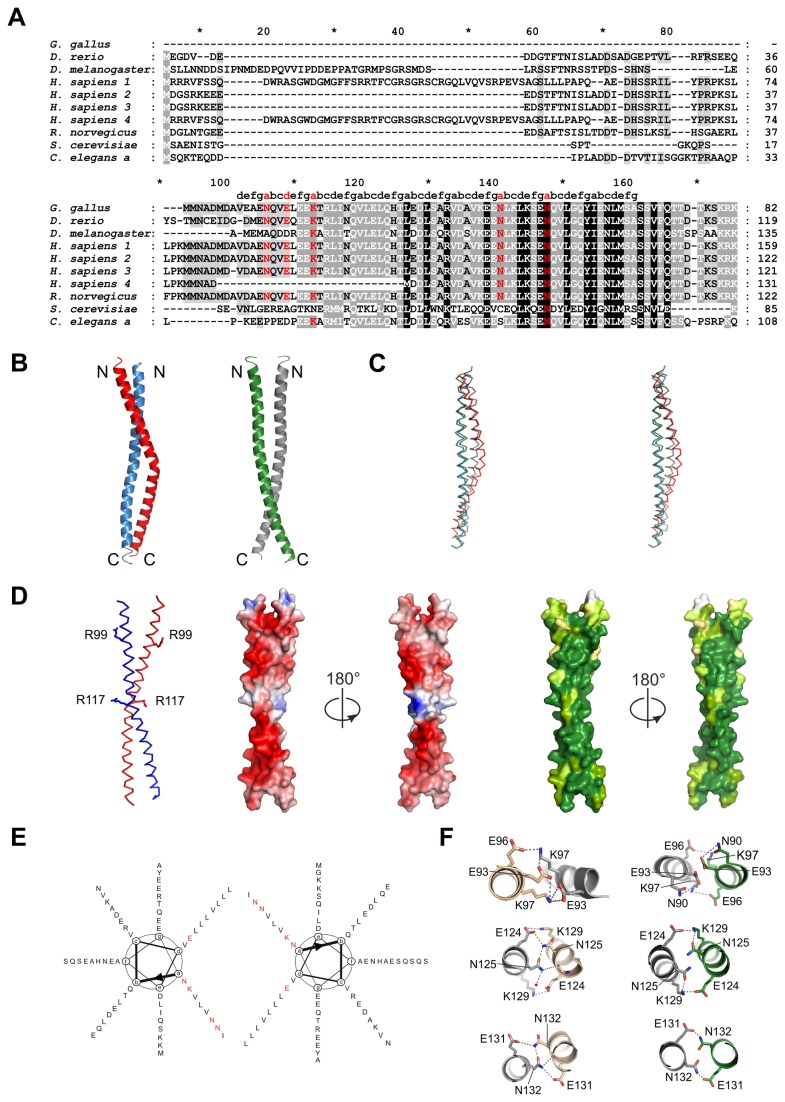
Structure of the SCOC coiled coil domain. (**A**) Sequence alignment of human SCOC isoforms and homologues was done with T-Coffee [35]. Accession numbers are: *G. gallus*: NP_001108324.1, *D. rerio*: NP_001071027.1, *D. melanogaster*: NP_651552.1, *H. sapiens* 1: NP_001146956.1, *H. sapiens* 2: Q9UIL1-2, *H. sapiens* 3: Q9UIL1-3, *H. sapiens* 4: Q9UIL1-4, *R. norvegicus*: NP_001013253.1, *S. cerevisiae*: NP_796378.3, *C. elegans* *a*: NP_001022752.1. Coiled coil heptad positions were assigned with TWISTER [29]. Non-canonical polar amino acids at core positions *a* and *d* are colored red. (**B**) Cartoon representation of the two SCOC dimers AB and CC’. Molecule A is colored red, B blue, C green and C’ grey. (**C**) Stereo view of dimers overlay. Same coloring as in (B). (**D**) Left panel shows a ribbon representation of dimer AB. Residues R99 and R117 are drawn as stick models. Electrostatic surface potential is shown for dimer AB in two orientations. Blue shows positive charge and red corresponds to negative charge. The first figure of the electrostatic surface potential is shown in the same orientation as the ribbon representation. The two right panels show the surface conservation of SCOC. Dark green correspond to strong conservation and lighter green shades represent less conserved regions. The first conservation figure is shown in the same orientation as the ribbon representation. Sequence alignments were done with T-Coffee and analysis of the degree of conservation was done with AMAS [36]. (**E**) Helical wheel diagram of the SCOC dimer. Polar and charged core residues are marked red. (**F**) Detailed views on molecular interactions of the non-canonical polar core positions of dimer AB (left panel) and dimer CC’ (right panel). Core residue E93, K97, N125 and N132, which were used for mutagenesis studies are stabilized through a network of hydrogen bonds and salt bridges as shown. Figures were prepared with PyMol [22].

At least four different human SCOC isoforms are known. Their N-termini are variable, whereas the approx. 70 residues long coiled coil domain is highly conserved, also among different species, highlighting its functional importance (Figure 1A).

Coiled coils are formed by at least two α-helices that are wound around each other forming a superhelical structure as reviewed in [12]. They are characterized by a heptad repeat pattern (*a,b,c,d,e,f,g*)_n_, where positions *a* and *d* are occupied by mostly apolar amino acids like leucine, valine and isoleucine [13]. These residues form the hydrophobic core of coiled coils. The nature of the amino acids at the *a/d*-positions is important for determining the oligomerisation state of a coiled coil protein [14-16]. Besides facilitating protein homo-oligomerisation, coiled coils are also very important for mediating protein-protein interactions. The coiled coil interaction network in *S. cerevisiae* was characterized through yeast two-hybrid assays. In the study of Wang et al., 3495 pairwise interactions were identified among 598 predicted coiled coil regions in 453 proteins, which are extensively involved in the organization of the cellular machinery [17].

SCOC-FEZ1 complex formation is also mediated through the coiled coil domains of SCOC and FEZ1 [2-4]. Here we present the crystal structure of the SCOC coiled coil domain as a first step towards the structural characterization of the SCOC-FEZ1 complex. SCOC is a dimeric coiled coil protein with an unusual high incidence of polar and charged residues at half of the heptad *a*-positions. Using mutagenesis studies we demonstrate that these residues are important for dimerization of SCOC. We further show that SCOC forms a homogeneous stable complex with the coiled coil domain of FEZ1 and that dimerization of SCOC is essential for this interaction.

## Materials and Methods

### Purification and crystallization of SCOC

SCOC(78-159) (SwissProt entry Q9UIL1 isoform1) was cloned in pET28a (Novagen) using NcoI and XhoI cleavage sites. A synthetic gene (Mr Gene) optimized for *E. coli* expression was used as a template for PCR. The sequence of the synthetic SCOC gene and all primers used in this study are listed in Table 1. SCOC(78-159) mutants were prepared with the QuikChange II Site-Directed Mutagenesis Kit (Stratagene). All SCOC constructs were purified with the same protocol. The plasmid was transformed into BL21 (DE3). LB medium was inoculated with an overnight preculture at 1:150 dilution. Cultures were grown at 37 °C in LB medium supplemented with 30 mg/L kanamycin for approx. 2 h until OD_600_ reached 0.5, and then expression was induced with 1 mM IPTG. After 3 hours of expression at 37 °C, bacteria were harvested with a JS-4.2 rotor in a Beckman J6-MI centrifuge at 4500 × g, 4 °C for 20 min. The pellet was resuspended in 500 mM NaCl, 50 mM HEPES pH 7.5. Cells were lysed in a microfluidizer M-110L (Microfluidics Corporation) and spun with a SS34 rotor in a Thermo Scientific Sorvall RC 6 PLUS centrifuge at 25000 × g and 4 °C. Supernatant was loaded onto a 5 ml StrepTrap HP column (GE Healthcare) using 500 mM NaCl, 50 mM HEPES pH 7.5 as running buffer. After washing with 35 ml buffer the protein was eluted with 30 ml buffer supplemented with 2.5 mM desthiobiotin. The protein was concentrated and applied onto a HiLoad Superdex 75 16/60 column (GE Healthcare) using 250 mM NaCl, 20 mM HEPES pH 7.5 as gel filtration buffer. The yield from a 10 L culture is 15 mg purified SCOC.

**Table 1:** Construct and primer list.

Strep-SCOC ccd M78 NcoI fwd	gaattccatatgatgaatgccgacatggatg
Strep-SCOC rev 1	ctccagctgccgcgcggcaccagtttacgtttgg
Strep-SCOC XhoI rev 2	ccgctcgagttatttttcgaactgcgggtggctccagctg
SCOC-Gateway ccd M78 fwd	caccatgatgaatgccgacatggatgc
SCOC-Gateway rev	tttacgtttggatttggtatcggtggtttga
SCOC L105M fwd	acccgtctgatcaaccaagttatggagctgcag
SCOC L105M rev	ctgcagctccataacttggttgatcagacgggt
SCOC R99E fwd	gtggaactggaggaaaaaaccgagctgatcaaccaagttctggag
SCOC R99E rev	ctccagaacttggttgatcagctcggttttttcctccagttccac
SCOC R117E fwd	actggaagatctgtctgccgaggtcgatgccgtaaaagagg
SCOC R117E rev	cctcttttacggcatcgacctcggcagacagatcttccagt
SCOC E93V fwd	tgccgaaaatcaggtggtactggaggaaaaaaccc
SCOC E93V rev	gggttttttcctccagtaccacctgattttcggca
SCOC E93V /K97L fwd	gccgaaaatcaggtggtactggaggaattaacccgtctgatcaac
SCOC E93V /K97L rev	gttgatcagacgggttaattcctccagtaccacctgattttcggc
SCOC N125L fwd	gtgtcgatgccgtaaaagaggagctactgaaactgaaaagtgagaatca
SCOC N125L rev	tgattctcacttttcagtttcagtagctcctcttttacggcatcgacac
SCOC N132V fwd	gaatctgaaactgaaaagtgaggttcaagtgctgggccagtatatc
SCOC N132V rev	gatatactggcccagcacttgaacctcacttttcagtttcagattc
FEZ1 ccd M227 NdeI fwd	ggaattccccatatgtctgggtctgag
FEZ1 ccd L290 XhoI rev	ccgctcgaggctgccgcgcggcaccagctttttcatcagttctcgctg
synthetic gene of SCOC isoform 1 full length	atgcgtcgccgtgtgttttctagccaggattggcgtgcttcaggatgggacggtatgggcttttttagtcgtcgtaccttctgtggtcgttcaggtcgttcttgtcgtggacagctggttcaagtttctcgcccggaagtttctgctggttctctgctgctgcctgctccacaagccgaagatcatagcagtcgtattctgtatcctcgcccaaaatccctgctgccgaaaatgatgaatgccgacatggatgccgttgatgccgaaaatcaggtggaactggaggaaaaaacccgtctgatcaaccaagttctggagctgcagcatacactggaagatctgtctgcccgtgtcgatgccgtaaaagaggagaatctgaaactgaaaagtgagaatcaagtgctgggccagtatatcgaaaatctgatgagcgcctctagcgtctttcaaaccaccgataccaaatccaaacgtaaataa
C-terminal Strep-tagged SCOC(78-159) construct, SCOC sequence is underlined	MAMMNADMDAVDAENQVELEEKTRLINQVLELQHTLEDLSARVDAVKEENLKLKSENQVLGQYIENLMSASSVFQTTDTKSKRKLVPRGSWSHPQFEK

Selenomethionine-labelled L105M SCOC(78-159) was expressed in minimal medium supplemented with selenomethionine in *E. coli* as described [18] and purified as the native protein with gel filtration buffer containing 2 mM TCEP. Purified proteins were concentrated, flash-frozen in liquid nitrogen, and stored at −80 °C.

For *in situ* proteolysis crystallization 3 mg/ml of selenomethionine-labelled L105M SCOC(78-159) were mixed with subtilisin in a 1:2000 (w/w) ratio and kept on ice until pipetting of the crystallization plates. Crystals were grown in hanging drops using Linbro plates at 20 °C by mixing 1 µl protein with 1 µl of the precipitant containing 100 mM sodium acetate pH 4.6, 0.7 M 1,6-hexanediol and 10 mM CoCl_2_. Crystals were soaked in mother liqueur supplemented with 20% PEG 400 and flash cooled in liquid nitrogen.

### Structure determination

X-ray diffraction data were collected at a wavelength of 0.9793 Å at 100 K at beamline X10SA (Swiss Light Source, Paul Scherrer Institute, Villigen, Switzerland). Data were processed and scaled with the XDS software package [19] (Table 2). The structure was solved by single-wavelength anomalous diffraction phasing with a dataset from a selenomethionine-labelled Strep-tagged L105M SCOC (78-159) crystal. Phasing and initial model building was done with Phenix [20]. Three of six Se sites from three molecules in the asymmetric were found, yielding an initial map with a Bayesian overall correlation coefficient of 39.0.

**Table 2:** Data collection and refinement statistics.

	**SCOC_78-159_ L105M**
*PDB entry*	4bwd
*Data collection*	
Space group	C222_1_
*Cell dimensions*	
a, b, c (Å)	71.0, 114.8, 93.3
α, β, γ (°)	90, 90, 90
Resolution (highest res. shell) (Å)	40-2.70 (2.8-2.70)
R_meas_	7.2 (57.7)
no. of observed reflections /unique reflections	69253/10743
	(6565/1019)
I/σ (I)	17.3 (3.7)
Completeness (%)	99.5 (96.4)
Wilson B factor (Å^2^)	58.7
Molecules/AU	3
*Refinement*	
R_work_/R_free_	21.9/26.0
*No of atoms total*	1424
residues included in model (number of protein atoms)	A: 86-146 (477)
	B: 88-147 (463)
	C: 86-146 (441)
Water *B-factors (Å^2^)*	43
Overall	52.1
Protein	52.0
Water	55.1
*r.m.s.d.*	
Bond lengths (Å)	0.008
Bond angles (°)	1.00

Coot was used for manual model building [21]. Refinement was performed with Phenix. The final model comprising residues 86-147 and structure factors were deposited in the PDB with accession code 4bwd. Figures were prepared with PyMOL [22]. Superimpositions of structures were done with LSQKAB from the CCP4 software suite [23,24].

### Purification of SCOC-FEZ1 complexes

Human FEZ1(227-290) was cloned with NdeI and XhoI restriction sites into vector pET22b (Novagen) using full length FEZ1 as template for PCR [9]. Both FEZ1(227-290) pET22b and SCOC(78-159) pET28a were co-transformed into BL21 (DE3) by electroporation.

Cultures were grown in LB medium supplemented with 100 mg/L ampicillin and 30 mg/L kanamycin. LB medium was inoculated with an overnight preculture at 1:150 dilution. Protein expression was induced with 1mM IPTG at an OD_600_ of approx. 0.5. Cells were harvested after further incubation for 3 h at 37 °C using a JS-4.2 rotor in a Beckman J6-MI centrifuge at 4500 × g, 4 °C for 20 min. After resuspending into 250 mM NaCl, 50 mM HEPES pH 7.5 cells were lysed in a microfluidizer M-110L (Microfluidics Corporation). Cell debris was removed by centrifugation at 25000 × g and 4 °C with SS34 rotor in a Thermo Scientific Sorvall RC 6 PLUS centrifuge. The supernatant was applied onto a 5 ml StrepTrap HP column. After washing with 35 ml buffer the complex was eluted with 30 ml buffer supplemented with 2.5 mM desthiobiotin. The complex was further purified on a Superdex 75 16/60 column using 250 mM NaCl, 50 mM HEPES pH 7.5 as running buffer. After concentration, the complex was flash-frozen in liquid nitrogen and stored at −80 °C. From a 5 L culture 2-3 mg FEZ1-SCOC complex were isolated.

### Size exclusion chromatography coupled with multi-angle laser light scattering (SEC-MALLS)

500 µL of 1.5 mg/ml sample were loaded manually onto a Superdex 200 10/300 GL connected to an Eclipse 2 system from Wyatt Technology with a DAWN EOS multi-angle light scattering setup and an Agilent 1100 series HPLC pump. After starting data acquisition, the sample was injected manually. This resulted in a shift of retention times for different measurements of the same protein, which had no influence on the determined molecular weight. Scattering data were analyzed with the manufacturer’s ASTRA software. Molecular weights were determined from three SEC-MALLS experiments for each protein.

### Mass spectrometry

Protein complexes were buffer-exchanged against 200 mM ammonium acetate using Micro Bio-spin 6 columns (Bio Rad). Mass spectra were acquired on an LCT mass spectrometer (Waters) modified for high masses [25] using in-house prepared gold coated glass capillaries [26]. Optimized instrument parameters were as follows: capillary voltage 1.7 kV, cone voltage 70 V, extractor 5 V and source backing pressure 5.77 mbar. Spectra were processed using MassLynx V4.1 (Waters). Complexes were assigned and complex masses were determined using in-house software Massign [27].

### Circular Dichroism (CD) spectroscopy

Measurements were done with a Chirascan Circular Dichroism spectrometer (Applied Photophysics) using a Hellma quartz cuvette with a path length of 0.1 cm. Samples were in a buffer consisting of 20 mM NaH_2_PO_4_ pH 7.5, 250 mM NaF. Far UV CD spectra of 15 µM SCOC were recorded between 200 and 260 nm with a step size of 0.5 nm, a bandwidth of 1.5 nm and an averaging time of 5 s at either 20 °C or 93 °C. Thermal melts were carried out from 20 °C to 93 °C at 208 nm with a heating rate of 0.5 °C/min. Bandwidth was 1.5 nm and the averaging time was 1.5 s.

Data were analyzed with the manufacturer’s ProView Software. Melting curves were fitted to a sigmoid shape and melting points were determined as the maxima of the first derivative of the function f(x) = Ab + ((At-Ab)/(1+exp((x0-x)/w))) + m. x0 is the transition point and corresponds to the melting temperature and w is the width of the sigmoidal slope. At (maximum ellipticity, corresponds to the unfolded protein) and Ab (minimum ellipticity, corresponding to the fully folded protein, axis intercept on Y-axis) describe the amplitude of the sigmoidal curve.

### Constructs and antibodies for in vivo studies

The open reading frames encoding human wild type or mutant SCOC(78-159) were amplified by PCR and inserted into the GATEWAY entry vector pENTR/D-TOPO. Eukaryotic plasmids expressing EmGFP-SCOC wild type or mutants were obtained by shuttling the inserts from their respective entry vectors to pcDNA6.2/N-EmGFP-DEST. The following antibodies were used in this study: anti-α-tubulin (Synaptic Systems), anti-LC3B (Novus Biologicals), anti-GM130 (BD Transduction Laboratories), anti-V5 (Santa Cruz Biotechnology), anti-green fluorescent protein (Synaptic Systems).

### Cell culture and transfection

Maintenance and transfection of HeLa SS6, PC-12 (clone 251) and human embryonic kidney (HEK) 293 cells were performed as previously described [9,28].

### Co-immunoprecipitations

One day after transfection, HEK 293 cells were lysed with ice-cold HNE buffer (50 mM HEPES, pH 7.2, 150 mM NaCl, 1% (v/v) Triton X-100, 1 mM EDTA) containing Complete EDTA-free protease inhibitor cocktail (Roche). Cell lysates were cleared by centrifugation with a table top centrifuge (10 000 × g, 10 min at 4 °C) and the resultant supernatant was incubated with anti-GFP antibodies for 3 h. Thirty microliters of protein G-Sepharose was subsequently added to the mixture and incubation continued for an additional hour. Immunoprecipitates were washed 3× with HNE buffer. Proteins were eluted with 2× LDS (lithium dodecyl sulfate (LDS) sample buffer) and analyzed by immunoblotting.

### Induction of autophagy and LC3-II lipidation assay

Constructs expressing different EmGFP-SCOC variants were individually transfected into PC12 cells using lipofectamine. Autophagy was induced a day after transfection by first washing the cells twice with serum-free DMEM and then maintaining them in serum-free DMEM for an additional 2 h at 37°C in 10% CO_2_. Cells were incubated in serum-free DMEM containing 100 nM Bafilomycin to block degradation of LC3-II. At the end of incubation, cells were washed twice with pre-warmed phosphate-buffered saline (PBS) and lysed with 200 µl of 2× LDS sample buffer. Lysates were sonicated for 15 min in an ultrasonic water bath (Sonorex RK100, Bandelin). After heating for 10 min at 70°C, lysates were subsequently passed through a tuberculin syringe fitted with a 23 gauge needle. 10 µl of each lysate was then analyzed by immunoblotting.

### Immunocytochemistry

HeLa cells were fixed with 3.6% paraformaldehyde in phosphate buffered saline (PBS, pH 7.3). Cells were then permeabilized with 0.3% Triton-X100 in PBS and blocked with 10% normal goat serum diluted in PBS. Coverslips were incubated with primary antibodies for 1 h. After washing, cells were incubated with Cy3-conjugated donkey anti-mouse antibodies (Jackson ImmunoResearch). Images were acquired using a ZEISS Axiovert 200M fluorescence microscope.

## Results

### Structure of the SCOC coiled coil domain

We determined the crystal structure of the coiled coil domain of human SCOC isoform 1 at 2.7 Å resolution (Figure 1 B, C). The structure was solved by single wavelength anomalous diffraction (SAD) phasing using selenomethionine labeled crystals of the L105M SCOC mutant. The final model comprises residues 86-147 (Table 2).

SCOC is a parallel left-handed coiled coil homodimer. The structure contains eight heptad repeats and has a length of about 80 Å. Intriguingly, we observed two distinct dimers in the crystal structure (Figure 1B). The asymmetric unit contains three SCOC molecules. Molecules A and B form dimer AB and the second dimer CC’ is composed of molecule C and a symmetry related copy of C. Dimer CC’ is a regular coiled coil, whereas chains A and B are not as tightly packed because molecule A is bent with a bulge around residues A116. Overlay of the two dimers showed that molecules B and C adopt a very similar conformation and superimpose with a r.m.s.d. of 1.0 Å for the Cα atoms (Figure 1C). In contrast, the differences between A and either B or C are more pronounced with r.m.s.d. values of 2.9 Å and 2.3 Å, respectively. The coiled coil pitch of dimer AB (residues 97-143) is 129 Å and 151 Å for dimer CC’ as calculated with the program TWISTER [29]. Both values are similar to the coiled coil pitch of 135 Å for the regular left-handed parallel coiled coil GCN4 leucine zipper dimer (pdb entry: 2ZTA).

The SCOC coiled coil domain (residues 78-146) has a calculated pI of 4.3 and the overall surface charge of the molecule is negative (Figure 1D). There are only a few conserved positively charged patches present, which includes residues R99 and R117. In general, the surface and core of SCOC are highly conserved, but residues at both ends and near the bulge of molecule A are more variable across species (Figure 1D).

Remarkably, half of the *a* heptad positions at the core of the coiled coil are occupied by polar and charged residues (Figure 1 A, E). Non-canonical polar pairings at *a-*positions are found at the N-terminal end of the coiled coil (N90 and K97) and close to the bulge of molecule A (N125 and N132) (Figure 1C). Additionally, there is one charged *d*-residue (E93), whereas the other *d*-positions are occupied by leucines, V121 and V86. However, V86 is localized at the beginning of the coiled coil domain, where the two subunits are still converging to form the coiled coil domain. The distance between the two V86 Cα atoms in dimer CC’ is 9.8 Å. The first ordered residue in subunit B is residue A88 so we cannot determine this value for dimer AB. For comparison the Cα distances for V121 in the central region of the coiled coil are 6.7 Å for dimer CC’ and 7.2 Å in dimer AB.

The charged and polar residues at the coiled coil interface are stabilized through salt bridges and hydrogen bonds, respectively. For example, E93 forms both intra- and intermolecular salt bridges with K97 in dimer AB and N125 forms a net of hydrogen bonds with E124 and K129 (Figure 1 F). Importantly, all polar and charged core residues are highly conserved among human isoforms and other species (Figure 1A). We expected that these residues result in weakened core interactions, creating a destabilized dimer, which would explain the observed conformational flexibility of SCOC. In order to test the influence of these polar core residues on the stability of the protein we generated two double core mutants E93V/K97L and N125L/N132V.

Additionally, a second set of mutants was prepared to probe SCOC-FEZ1 complex formation. SCOC interacts with the coiled coil domain of FEZ1 [2-4]. The FEZ1 coiled-coil domain (residues 227-290) is negatively charged with a calculated pI of 4.7. We therefore speculated that SCOC residues R99 and R117, which are conserved and surface exposed might be important for SCOC-FEZ1 complex formation and prepared the R99E and R117E SCOC mutants.

### Characterization of SCOC mutants

**Figure 2:**
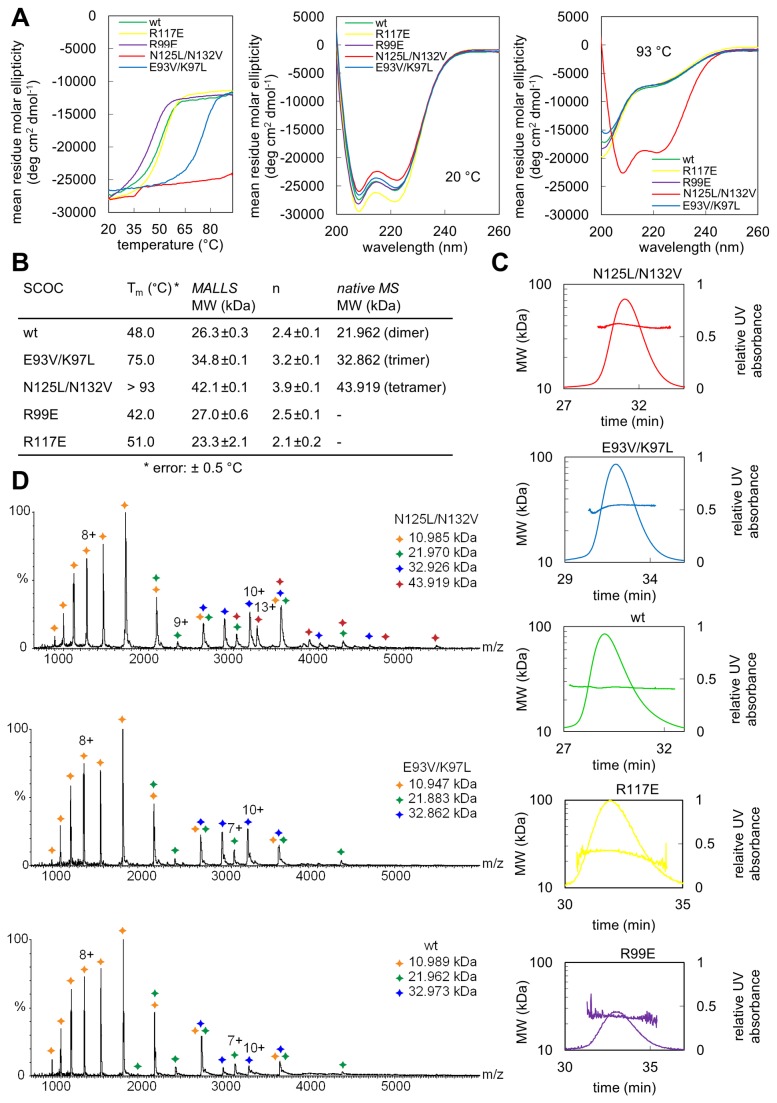
Biophysical characterization of SCOC mutants. (**A**) Analysis of SCOC mutants by CD spectroscopy. Thermal unfolding curves were recorded from 20 to 93 °C at a wavelength of 208 nm, which is the global minimum in the SCOC CD spectrum (left panel). The protein concentration was approx. 15 µM. Middle and right panels show CD spectra of SCOC mutants measured from 200 to 260 nm at 20 °C and 93 °C, respectively. (**B**) Summary of the melting temperatures (T_m_) and molecular weights (MW) measured for all SCOC constructs. Molecular weights were determined from three SEC-MALLS measurements for each mutant. The stoichiometry (n) is the ratio of the measured molecular weight by MALLS and the MW of a single Strep-tagged SCOC molecule (11206 Da). Molecular weights for the wild-type and double core mutants were also determined by native mass spectrometry. (**C**) Elution profiles and corresponding molecular weights determined by SEC-MALLS are shown for wild-type SCOC and mutants. (**D**) Mass spectra of native wild-type SCOC and the E93V/K97L and N125L/N132V mutants are shown.

We used far-UV circular dichroism (CD) spectroscopy to analyze the stabilities of wild-type SCOC and its mutants. Thermal unfolding curves were measured between 20 °C and 93 °C at a wavelength of 208 nm (Figure 2A). Wild-type SCOC(78-159) unfolds at 48 °C. Both arginine surface mutations had no significant effect on the stability of the proteins (Figure 2A, B). In contrast, both double core mutants are much more stable than the wild-type protein. The melting temperature of E93V/K97L is increased by almost 30 °C. Strikingly, the N125L/N132V mutant is extremely stable and remained folded at even 93 °C (Figure 2A).

We speculated that these dramatic differences in stability of both core double mutants might be due to a change of oligomerisation state. We therefore used size exclusion chromatography coupled with multi-angle laser light scattering (SEC-MALLS) to measure the molecular weights of the mutant proteins. The sequence-based molecular weight of Strep-tagged SCOC is 11.2 kDa. The measured molecular weight of wild-type SCOC is 26.3±0.3 kDa, which indicates a dimeric oligomerisation state as observed in the crystal structure (Figure 2B, C). The molecular weight determined for N125L/N132V is 42.1±0.1 kDa, which corresponds to a tetramer. The E93V/K97L double mutant forms a trimer (34.8±0.1 kDa). Both R99E and R117E surface mutants are dimers (Figure 2C).

We also analyzed the two double core mutants and the wild-type protein with native mass spectrometry (Figure 2B, D). Spectra of intact complexes were recorded employing a mass spectrometer modified for transmission of high mass complexes [25]. Peak series observed indicated the presence of dimeric (wild-type), trimeric (E93V/K97L) and tetrameric (N125L/N132V) SCOC. Monomeric SCOC as well as lower assembly states of the respective complexes were observed in all spectra presumably due to in-solution dissociation in ammonium acetate buffer. Masses of 10.97, 21.93, 32.92 and 43.92 kDa were determined for the monomer, dimer, trimer and tetramer, respectively. Small amounts of trimer were also observed in the wild-type sample, however, the intensity of the trimer was significantly increased for the E93V/K97L mutant.

**Figure 3:**
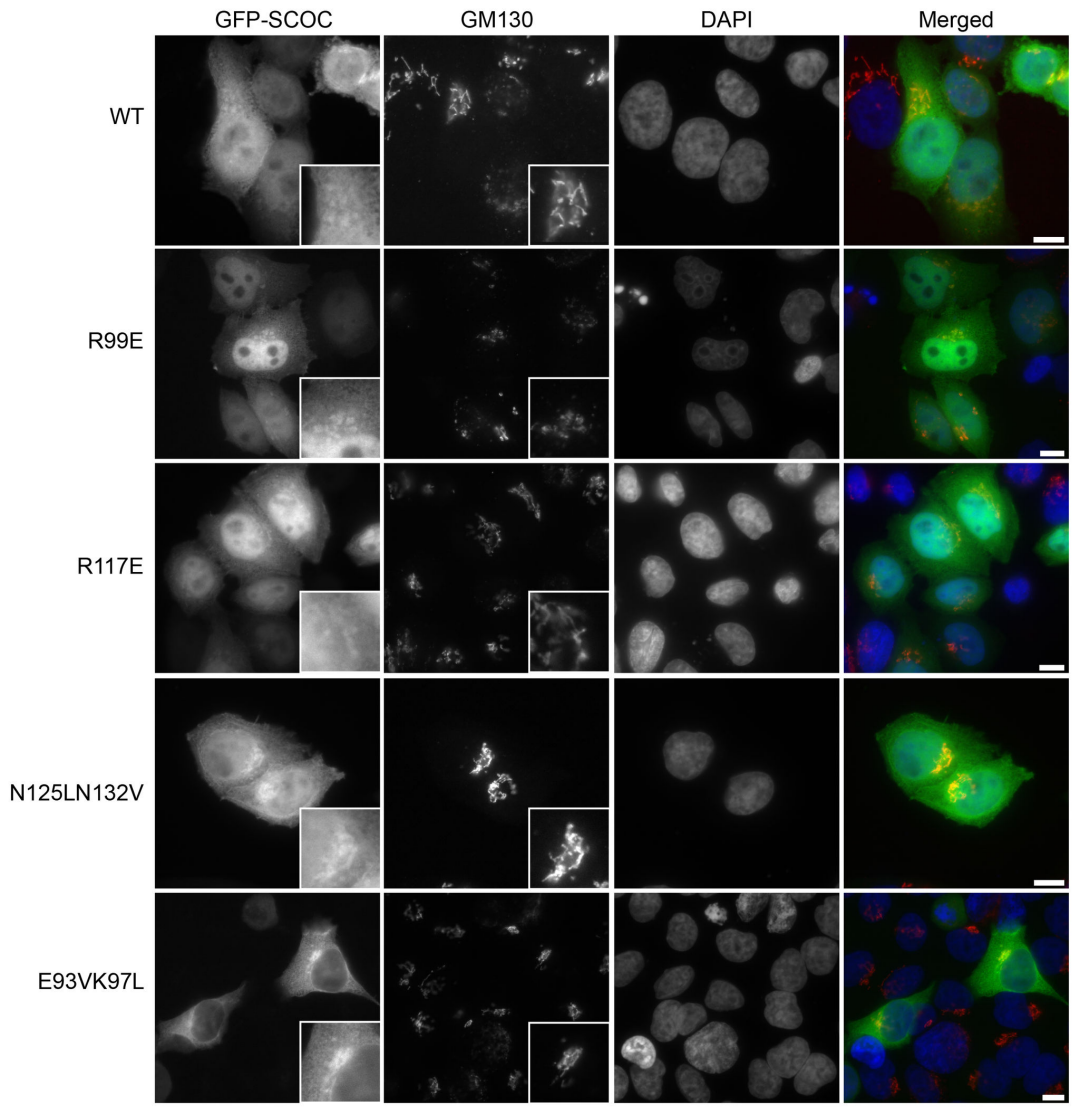
Intracellular distribution of EmGFP-SCOC in HeLa cells. EmGFP-SCOC(78-159) variants transiently expressed in HeLa cells show partial localization with Golgi compartments as visualized by co-staining with GM130. Wild-type SCOC (WT), SCOC(R99E), SCOC(R117E) and SCOC(N125V/N132L) are diffusely distributed in both cytosol and nucleus. SCOC(E93V/K97L) localizes exclusively in the cytosol. Scale bar, 10 µm.

In order to analyze whether the different oligomerisation states of the core mutants had an impact on the cellular localization of SCOC, we transfected N-terminal tagged EmGFP-SCOC constructs into HeLa cells. All mutants and wild-type SCOC showed partial co-localization with the Golgi (Figure 3). The EmGFP-SCOC constructs localized to both cytoplasm and nucleus, except the E93V/K97L trimer that showed only cytoplasmic localization. Since SCOC was recently identified as a positive regulator of autophagy [2], we performed a LC3 lipidation assay with the SCOC mutants to assess their effects on autophagy. During autophagy, the C-terminus of LC3-I is conjugated to phosphatidylethanolamine, resulting in formation of lipidated LC3-II that is targeted to autophagosomal membranes. Induction of autophagy (e.g. under conditions of nutrient starvation) is accompanied by increased levels of LC3-II whereas inhibition of autophagic induction prevents such an increase. These two forms of LC3 are easily resolved and detectable on immunoblots. Thus, changes in autophagy can be monitored by documenting fluctuations in LC3-II versus LC3-I [30].

**Figure 4:**
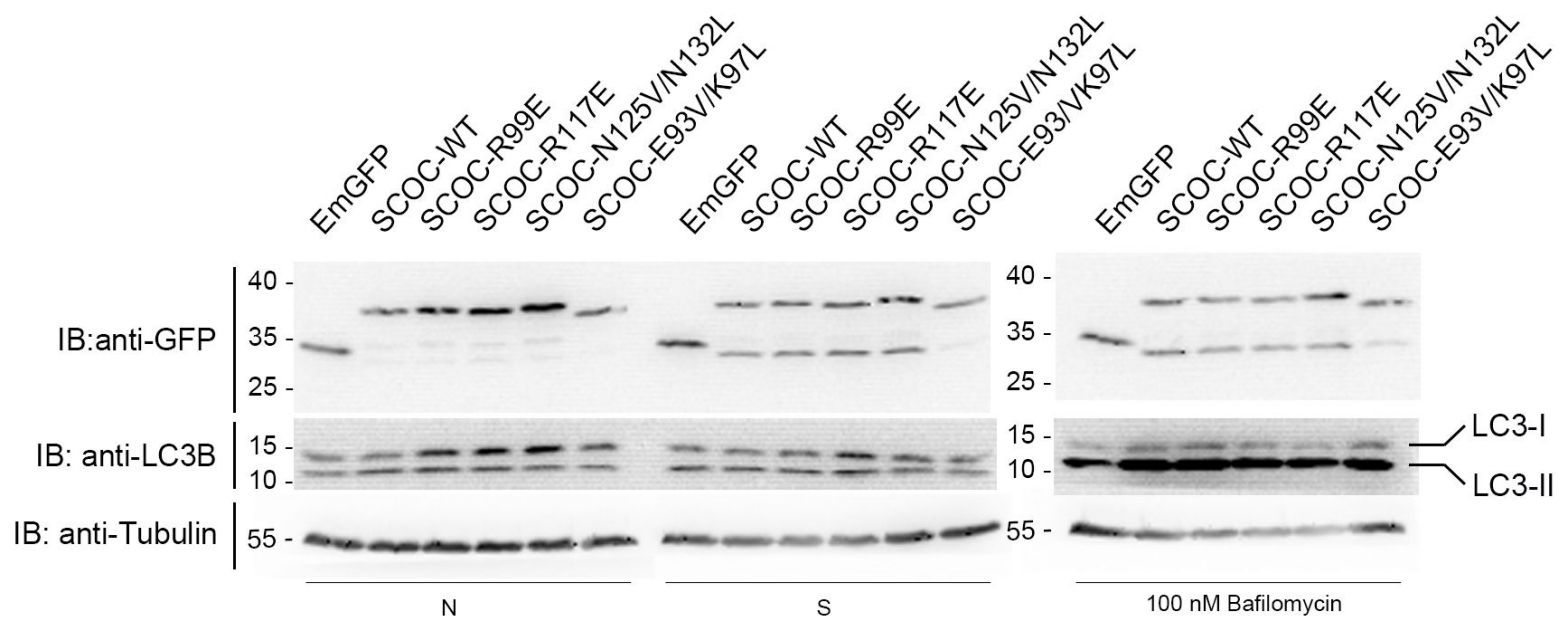
LC3-II lipidation assays with SCOC mutants. Overexpression of SCOC mutants does not increase LC3-II levels. LC3-II lipidation was monitored by immunoblotting of lysates obtained from PC12 cells expressing either EmGFP alone or EmGFP-SCOC variants. Transfected cells were either maintained in nutrient conditions (N) or starved for 2 hours (S) to induce autophagy. In addition, inhibition of LC3 degradation after autophagic induction was effected by the addition of 100 nM bafilomycin. A smaller fragment recognized by the GFP antibody was detected during starvation in all EmGFP-SCOC constructs, apart from the E93V/K97L mutant. However, basal autophagy appears unaffected by this degradation product because increased levels of LC3-II were observed in cells treated with bafilomycin where LC3-II turnover is blocked.

Under both nutrient-rich and starvation conditions, over-expression of all SCOC variants in PC12 cells did not cause significant changes in the ratio of LC3-II versus LC3-I as compared to control cells transfected with a plasmid expressing only EmGFP (Figure 4). Addition of 100 nM bafilomycin, an inhibitor of LC3-II degradation by preventing fusion of autophagosomes and lysosomes [31], also revealed no significant changes in LC3-II levels in cells transfected with SCOC variants over control cells. Thus, under the conditions tested here, SCOC does not affect autophagy. Next, we analyzed FEZ1 binding properties of the SCOC mutants.

### Analysis of SCOC-FEZ1 complex formation

**Figure 5:**
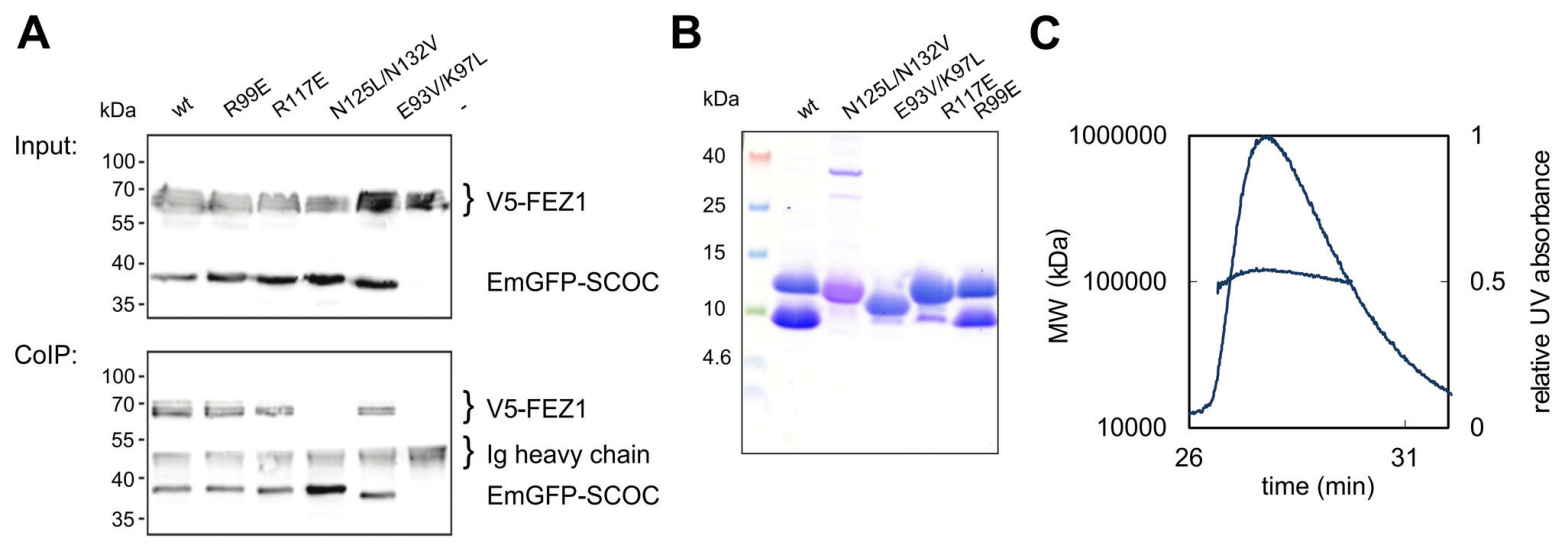
Analysis of SCOC-FEZ1 complex formation. (**A**) Co-immunoprecipitation of full length FEZ1 tagged N-terminally with a V5 epitope and various EmGFP-SCOC(78-159) variants from transfected HEK 293 cell lysates using an anti-GFP antibody. (**B**) Co-expression of Strep-SCOC(78-159) constructs with His_6_-FEZ1(227-290). Samples eluting from a StrepTrap column were analyzed with a Coomassie stained Schägger gel. (**C**) SEC-MALLS measurement of wild-type SCOC(78-159)-FEZ1(227-290) complex. The concentration of the complex ranged from 1 to 1.5 mg/ml for SEC-MALLS measurements.

We first performed co-immunoprecipitation experiments with full-length FEZ1 and EmGFP-SCOC constructs expressed in HEK 293 cells (Figure 5A). The tetrameric core mutant N125L/N132V did not bind FEZ1, whereas the other mutants formed a complex *in vivo*.

The coiled coil region of FEZ1 binds SCOC as previously shown by yeast two hybrid assays, co-immunoprecipitation and Blue-native–PAGE [2-4]. Therefore, we also tested binding of this domain to our SCOC construct. A FEZ1cc construct comprising residues 227-290 was not soluble when expressed alone, therefore we co-expressed His-tagged FEZ1cc and Strep-tagged SCOC(78-159) in *E. coli*. Both proteins co-purified from a StrepTrap column and gel filtration column. SEC-MALLS measurements yielded a molecular weight of 120.2±4 kDa for the SCOC-FEZ1cc complex and showed that the complex is homogeneous (Figure 5B, C). FEZ1 is a dimer in solution [3,5,32]. Assuming that both proteins are dimers and that they interact with a 1:1 stoichiometry, there would be six copies of each protein in the SCOC-FEZ1 complex.

We also co-expressed FEZ1cc with the SCOC mutants. The tetrameric N125L/N132V and trimeric E93V/K97L mutants did not bind FEZ1cc showing that SCOC dimerization is crucial for SCOC-FEZ1cc complex formation. R117 is required for FEZ1cc interaction, because binding of R117E to FEZ1cc was almost completely abolished. The second arginine R99 is not important for complex formation, since R99E still interacted with FEZ1cc (Figure 5B).

However, both R117E and the trimeric SCOC mutant also bound full-length FEZ1 in our co-immunoprecipitation experiments. Based on these results we cannot exclude that the N-terminal region of FEZ1 might also be involved in SCOC binding, which was in fact reported earlier for NEK1 (Nima-related kinase 1). The coiled coil region of NEK1 comprising residues 497-555 interacts with both the coiled-coil region of FEZ1 and the N-terminal region of FEZ1 [33].

## Discussion

In this study we determined the structure of the SCOC coiled coil domain. We observed conformational flexibility of the SCOC dimer as evident by the occurrence of two distinct dimers in the crystal structure. The largest difference between the two dimers is the bulge around residues A116 in subunit A (Figure 1 B,C). This difference is not due to crystal packing contacts because only the N- and C-termini of the three molecules in the asymmetric unit interact with symmetry-related molecules.

Instead, we explain this plasticity with the enrichment of polar and charged residues at the *a/d*-heptad positions. Half of the *a*-positions and one of the *d*-positions (E93) in SCOC are occupied with polar and charged amino acids. Hydrophobic core packing is a key determinant for the stability of a coiled coil protein and polar residues at the core have a destabilizing effect and influence the oligomerization state of a coiled coil protein [15]. The influence of different amino acids at *a/d* heptad positions on the oligomerisation state and stability of a coiled coil protein was studied with the GCN4 leucine zipper in a landmark publication [14]. The GCN4 leucine zipper is a parallel two-stranded coiled coil with a single polar core residue (N16) at an *a*-position [34]. When N16 is replaced with a valine, the resulting mutant forms a mixture of dimers and trimers [14]. The N16V GCN4 leucine zipper mutant is much more stable with a melting temperature of 95 °C than the wild-type protein, which unfolds at 53 °C [14]. The authors concluded that N16 imposes specificity for dimerisation of the GCN4 leucine zipper at the expense of its stability. Our results are in agreement with these data. Replacement of the polar and charged *a/d* heptad residues in SCOC with either leucines or valines led to a dramatically increased stability of both double core mutants. The oligomerisation states of these mutants changed to either trimer (E93V/K97L) or tetramer (N125L/N132V) as shown by SEC-MALLS and native mass spectrometry measurements. In the mass spectrometry spectrum of wild-type SCOC also trimer peaks were observed, however with significantly decreased intensities in comparison with the trimeric E93V/K97L mutant. Although we cannot exclude that a small portion of wild-type SCOC is trimeric in solution, dimerisation of SCOC is consistent with the SEC-MALLS data, its thermal stability and the crystal structure. The presence of the C-terminal Strep-tag in the recombinant proteins used in this study is unlikely to affect SCOC oligomerization because the tag itself does not oligomerize. Also, although the Strep-tag is not visible in the electron density map, the two subunits of the dimer diverge near their C-termini so that the Strep-tags are not in close proximity.

The SCOC-FEZ1 complex plays a regulatory role for induction and progression of starvation induced autophagy [2]. Deletion of the *C. elegans* SCOC homologue UNC-69 resulted in defects of axon growth, guidance and their fasciculation. Abnormal presynaptic organization was also observed, implying a function of the SCOC-FEZ1 complex in axonal transport of vesicles [4]. Here we demonstrated that SCOC and FEZ1cc form a stable homogeneous complex with a molecular weight of 120 kDa, which would correspond to six copies of each molecule assuming a 1:1 stoichiometry. We further showed that dimerization of SCOC is crucial for SCOC-FEZ1 complex formation, demonstrating the functional importance of the polar and charged core residues, which are needed for dimer formation of SCOC. We also found that the SCOC surface residues R117 is required for SCOC-FEZ1 binding.

Further structural characterization of the SCOC-FEZ1 complex will help us to gain new insights into how this complex fulfills its diverse functions.

## Accession Numbers

Coordinates and structure factors were deposited in the PDB with accession code 4bwd.
