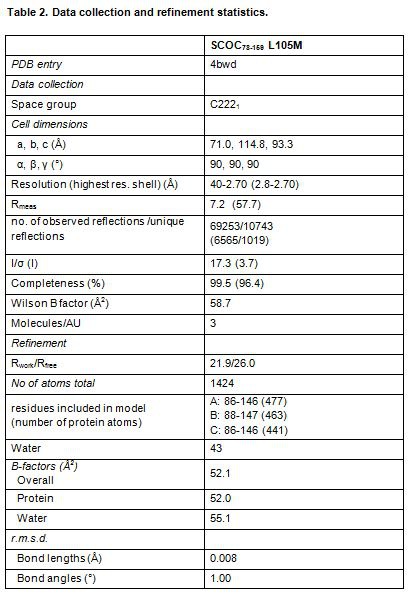# Correction: Crystal Structure of the Human Short Coiled Coil Protein and Insights into SCOC-FEZ1 Complex Formation

**DOI:** 10.1371/annotation/cb60d973-58aa-45e0-bbb3-1c7f5a42a8d2

**Published:** 2013-10-30

**Authors:** Caroline Behrens, Beyenech Binotti, Carla Schmidt, Carol V. Robinson, John Jia En Chua, Karin Kühnel

Due to issues with the typesetting process, there was an error in Table 2. The correct version of Table 2 is available here: 

**Figure:**